# Air pollution, fetal and infant tobacco smoke exposure, and wheezing in preschool children: a population-based prospective birth cohort

**DOI:** 10.1186/1476-069X-11-91

**Published:** 2012-12-11

**Authors:** Agnes MM Sonnenschein-van der Voort, Yvonne de Kluizenaar, Vincent WV Jaddoe, Carmelo Gabriele, Hein Raat, Henriëtte A Moll, Albert Hofman, Frank H Pierik, Henk ME Miedema, Johan C de Jongste, Liesbeth Duijts

**Affiliations:** 1 The Generation R Study Group, Erasmus Medical Center, Rotterdam, The Netherlands; 2 Department of Pediatrics, Division of Respiratory Medicine, Erasmus Medical Center, Rotterdam, The Netherlands; 3 Department of Epidemiology, Erasmus Medical Center, Rotterdam, The Netherlands; 4 Department of Urban Environment and Safety, Netherlands Organization for Applied Scientific Research (TNO), Delft, The Netherlands; 5 Department of Pediatrics, Erasmus Medical Center, Rotterdam, The Netherlands; 6 Department of Pediatric Pulmonology and Allergology, Wilhelmina Children’s Hospital, Utrecht, The Netherlands; 7 Department of Public Health, Erasmus Medical Center, Rotterdam, The Netherlands; 8 Department of Pediatrics, Division of Neonatology, Erasmus Medical Center, Rotterdam, The Netherlands

**Keywords:** Cohort study, Asthma, Pediatrics, Environmental tobacco smoke exposure, Air pollution

## Abstract

**Background:**

Air pollution is associated with asthma exacerbations. We examined the associations of exposure to ambient particulate matter (PM_10_) and nitrogen dioxide (NO_2_) with the risk of wheezing in preschool children, and assessed whether these associations were modified by tobacco smoke exposure.

**Methods:**

This study was embedded in the Generation R Study, a population-based prospective cohort study among 4,634 children. PM_10_ and NO_2_ levels were estimated for the home addresses using dispersion modeling. Annual parental reports of wheezing until the age of 3 years and fetal and infant tobacco smoke exposure was obtained by questionnaires.

**Results:**

Average annual PM_10_ or NO_2_ exposure levels per year were not associated with wheezing in the same year. Longitudinal analyses revealed non-significant tendencies towards positive associations of PM_10_ or NO_2_ exposure levels with wheezing during the first 3 years of life (overall odds ratios (95% confidence interval): 1.21 (0.79, 1.87) and 1.06 (0.92, 1.22)) per 10 μg/m^3^ increase PM_10_ and NO_2_, respectively). Stratified analyses showed that the associations were stronger and only significant among children who were exposed to both fetal and infant tobacco smoke (overall odds ratios 4.54 (1.17, 17.65) and 1.85 (1.15, 2.96)) per 10 μg/m^3^ increase PM_10_ and NO_2_, respectively (p-value for interactions <0.05).

**Conclusions:**

Our results suggest that long term exposure to traffic-related air pollutants is associated with increased risks of wheezing in children exposed to tobacco smoke in fetal life and infancy. Smoke exposure in early life might lead to increased vulnerability of the lungs to air pollution.

## Background

Higher exposure levels to air pollutants have been associated with increased risks of asthma exacerbations in adults and children aged older than 5 years [1-5]. The influence of air pollution on asthma and wheezing in younger children is less clear [6-9]. The effects of air pollutants on airway symptoms may differ between children and adults. Children older than 6 months of age may breathe more through the mouth than adults, and benefit less from the filtering, humidifying and temperature raising effect of the nose and might therefore inhale higher air pollutants levels [10]. Also, children spend more time outdoors than adults, and have a larger ratio of lung surface area to body weight [7,10,11], leading to a potential stronger effect of air pollution on airway symptoms, including wheezing [12]. A limited number of prospective birth cohort studies suggested associations of exposure to traffic-related air pollution, including particulate matter (PM_10_) and nitrogen dioxide (NO_2_), and the risk of wheezing and asthma in children up to the age of 8 years [8,9,13,14]. Thus far, results seem inconsistent [6]. This might be due to differences in study design, exposure and outcome assessment or confounding due to socio-demographic variables or a family history of asthma. Like some other environmental exposures, fetal and infant tobacco smoke exposure negatively influence the risk of asthma symptoms in early childhood, and might increase the susceptibility for the adverse effects of air pollution [15]. Therefore the associations between air pollution and asthma symptoms may be modified by tobacco smoke exposure [3].

We examined the associations of exposure to traffic-related air pollutants PM_10_ and NO_2,_ during different exposure windows, with the risk of wheezing in preschool children in a prospective birth cohort study among 4,634 children living in the city of Rotterdam, The Netherlands. In addition, we assessed whether fetal or infant tobacco smoke exposure modified these associations.

## Methods

### Design and setting

**Figure 1:**
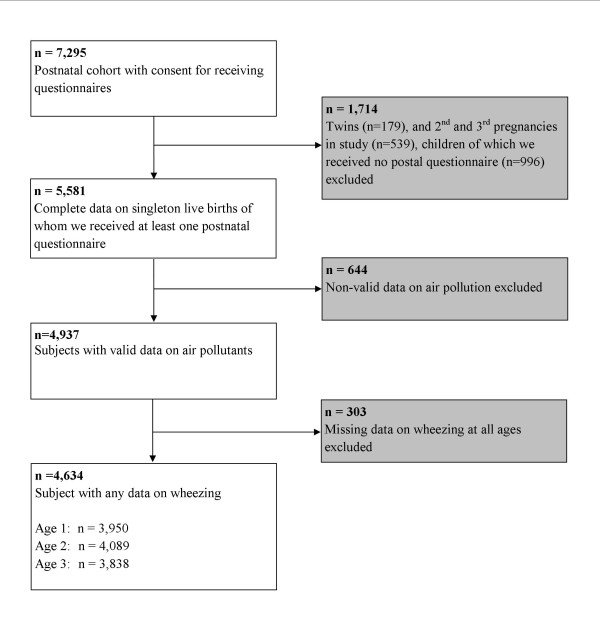
Flow chart of participants in study.

This study was embedded in the Generation R Study, a prospective cohort study from early fetal life to young adulthood in Rotterdam in the Netherlands [16]. The study protocol was approved by the Medical Ethical Committee of the Erasmus Medical Centre, Rotterdam. Written informed consent was obtained from all participants. In total 7,295 children born between 2002 and 2006 and their parents participated in the postnatal phase of the study. Of all eligible children in the study area, 61% participated in the present study. We excluded twins (n = 179), 2^nd^ and 3^rd^ pregnancies in the study (n = 539) and children of whom we did not receive any questionnaire (n = 996). Of the remaining children (n = 5,581) valid air pollution data were available for 4,937 children (Figure 1). Air pollution exposure could not be assessed for 644 children, due to incomplete address history, moving outside the study area or invalid measurements. We excluded children without any information about wheezing (n = 303 subjects). The final study population for analysis consisted of 4,634 children.

### Traffic-related air pollution exposure

Individual child exposures levels to particulate matter (PM_10_) and nitrogen dioxide (NO_2_) were assessed at the home address, using a combination of continuous monitoring and dispersion modeling, taking into account both the spatial and temporal variation in air pollution. The exposure assessment has been described in detail previously [17]. Briefly, annual average concentrations of PM_10_ and NO_2_ for the years 2002–2008 were assessed for all addresses in the study area. This was done using the 3 Dutch national standard methods for air quality modeling, designated to calculate the contribution of different air pollution sources [18]. Subsequently, hourly concentrations of PM_10_ and NO_2_ were derived, using air pollution measurements from 3 continuous monitoring stations (hourly calibration), taking into account wind conditions and fixed temporal patterns in source contributions. Based on participants’ home addresses, we derived individual exposure estimates for different periods during the first 3 years of life, including average exposure to air pollutants annually and overall. Average exposures were calculated for periods with <20% of the concentrations missing. For the other periods, air pollution exposures were set to missing. The performance of this model has been evaluated by two studies in the same study area which show a good agreement between predicted annual average PM_10_ and NO_2_ concentrations, and concentrations measured at monitoring stations [19,20].

### Respiratory symptoms

Information on wheezing (“Has your child had problems with a wheezing chest during the last year ?” no; yes) was obtained by questionnaires at the ages of 1, 2 and 3 years. Questions were adapted from the International Study on Asthma and Allergy in Childhood (ISAAC) [21]. Response rates for these questionnaires were 71%, 76% and 72%, respectively [22].

### Covariates

Information on maternal educational level, parity, smoking habits, smoking habits of the partner, history of asthma or atopy, children’s ethnicity and pet keeping were obtained by a questionnaire at enrolment. We used parity as a proxy for siblings (correlation: kappa = 0.894). Fetal smoke exposure was defined using data of maternal smoking habits during first, second and third trimester of pregnancy collected by questionnaires. We categorised groups as those children who were never exposed to tobacco smoke or in first trimester only (no fetal smoke exposure) and those who were continuously exposed to tobacco smoke in trimesters thereafter (fetal smoke exposure) [15]. Infant smoke exposure was defined as exposure to household tobacco smoke by anyone at the age of 2 years of the child (no; yes, data collected by questionnaires). Sex, gestational age at birth and birth weight of the children were obtained from midwife and hospital registries at birth. Postal questionnaires sent at the ages of 6 and 12 months provided information about breastfeeding. A questionnaire sent at the age of 12 months provided information on daycare attendance. Questionnaires filled in by the parents at the ages of 1, 2 and 3 years provided information about doctor attended lower respiratory tract infections (Has your child had pertussis, bronchitis, bronchiolitis or pneumonia in the past year for which a doctor or hospital was attended? no; yes) [16,22].

### Statistical analysis

We used multiple logistic regression models to analyze the associations of exposure to air pollution in the previous year with the risks of wheezing at the ages of 1, 2 and 3 years. With Generalized Estimating Equation (GEE) analyses, we were able to take the correlation between repeated measurements in the same subject into account, and to calculate the overall effect (average air pollution levels in the first 3 years of life with wheezing at age 1 to 3 years combined). We used a compound symmetry corrlation matrix in these models. All models were adjusted for potential confounders including maternal age, education, parity, smoking habits during pregnancy, smoking habits of the partner, history of asthma or atopy, and children’s sex, gestational age at birth, birth weight, ethnicity, breastfeeding status, daycare attendance, pet keeping and lower respiratory tract infections. Average exposures to PM_10_ and NO_2_, annually and overall, were analyzed as continuous variables and as quartiles (lowest quartile as the reference group). Tests for trend were performed by including average air pollutant concentration levels as continuous variables into the fully adjusted logistic regression model and we calculated the risk per 10 μg/m^3^ increase. Next, we stratified our models for tobacco smoke exposure to assess whether any observed association of air pollution with childhood wheezing was modified by environmental tobacco smoke exposure. For this analysis we also tested the interaction between air pollution and environmental tobacco smoke exposure. The tobacco smoke variables were combined into a new variable with 4 early smoke exposure categories: never; only fetal; only infant; and fetal and infant, using the variables about maternal smoking habits during pregnancy (fetal smoke exposure) and exposure to household tobacco smoke at the age of 2 years (infant smoke exposure). We performed multiple imputations to handle missing values of the covariates and outcomes by generating 25 independent datasets [23]. We imputed both covariates and outcomes, as missing values may introduce bias in GEE models [24]. Imputations were based on the relationships between all covariates and outcomes included in this study plus paternal age, educational level, history of asthma or atopy and information about shortness of breath in the past year of the children at the age of 1, 2 and 3 years. All datasets were analyzed separately after which results were combined. No differences in results were observed between analyses with imputed missing data or complete cases only. We only present results based on imputed datasets. All measures of association are presented with their 95% confidence intervals (CI). Statistical analyses were performed using the Statistical Package of Social Sciences version 17.0 for Windows (SPSS Inc., Chicago, IL, USA) and SAS 9.2 (SAS institute, Cary, NC, USA).

## Results and discussion

### Subject characteristics

Children were born at a median gestational age of 39.9 (5-95% range: 37.0-42.1) weeks with a mean birth weight of 3,439 (SD 556) grams (Table 1). Of all children who were exposed to tobacco smoke during fetal life, 59.3% was exposed to household tobacco smoke in infancy, whereas of all children who were not exposed to tobacco smoke during fetal life, 12.2% was exposed to household tobacco smoke in infancy. (Additional file 1: Table S1). The wheezing prevalence declined with increasing age. Mean annual PM_10_ levels were 28.9, 28.3 and 27.9 μg/m^3^ and mean annual NO_2_ levels were 38.7, 37.5 and 36.2 μg/m^3^ at the ages of 1, 2 and 3 years, respectively (Additional file 1: Table S2).

**Table 1:** Maternal and child characteristics

	**n** = **4**,**634**	
	**Observed**	**After multiple imputations**
**Maternal characteristics**		
Age (years)*	31.1 (4.9)	31.1 (4.9)
Highest completed education (%)		
Non-completed, primary or secondary	47.1 (2,050)	48.2 (2,234)
Higher	52.9 (2,299)	51.8 (2,400)
*Missing*	*6*.*2* (*285*)	-
Parity (%)		
Nulliparity	61.6 (2,762)	61.4 (2,844)
Multiparity	38.4 (1,722)	38.6 (1,790)
*Missing*	*3*.*2* (*150*)	-
History of asthma or atopy (%)		
No	61.9 (2,369)	59.0 (3,734)
Yes	38.1 (1,460)	41.0 (1,900)
*Missing*	*17*.*4* (*805*)	-
**Fetal and Child characteristics**		
Male sex (%)	49.9 (2,313)	49.9 (2,313)
Gestational age at birth (weeks)^$^	39.9 (37.0-42.1)	39.9 (37.0-42.1)
Birth weight (grams)*	3,439 (556)	3,439 (556)
Ethnicity (%)		
European	70.4 (3,144)	69.9 (3,240)
Non-European	29.6 (1,320)	30.1 (1,394)
*Missing*	*3*.*7* (*170*)	-
Breastfed (%)		
No	7.7 (339)	8.0 (371)
Yes	92.3 (4,089)	92.0 (4,263)
*Missing*	*4*.*4* (*206*)	-
Day care attendance (%)		
No	48.0 (1,894)	50.0 (2,316)
Yes	52.0 (2,050)	50.0 (2,318)
*Missing*	*14*.*9* (*690*)	-
Pet keeping (%)		
No	65.5 (2,399)	64.6 (2,993)
Yes	34.5 (1,263)	35.4 (1,641)
*Missing*	*21*.*0* (*972*)	-
Lower respiratory tract infections age 1 year (%)		
No	86.4 (3,165)	85.4 (3,957)
Yes	13.6 (498)	14.6 (677)
*Missing*	*21*.*0* (*971*)	-
Lower respiratory tract infections age 2 years (%)		
No	87.9 (3,494)	87.4 (4,052)
Yes	12.1 (484)	12.6 (582)
*Missing*	*14*.*2* (*659*)	-
Lower respiratory tract infections age 3 years (%)		
No	93.3 (3,453)	92.7 (4,294)
Yes	6.7 (247)	7.3 (340)
*Missing*	*20*.*2* (*934*)	-
Smoking of father (%)		
No	57.4 (2,153)	57.4 (2,658)
Yes	42.6 (1,599)	42.6 (1,976)
*Missing*	*19*.*0* (*882*)	-
Fetal smoke exposure (%)		
No	86.9 (3,246)	86.4 (4003)
Yes	13.1 (489)	13.6 (631)
*Missing*	*19*.*4* (*899*)	-
Infant smoke exposure (%)		
No	82.3 (3,391)	81.4 (3,770)
Yes	17.7 (728)	18.6 (864)
*Missing*	*11*.*1* (*515*)	-
Wheezing age 1 year (%)		
No	74.0 (2,922)	74.1 (3,433)
Yes	26.0 (1,028)	25.9 (1,201)
*Missing*	*14*.*8* (*684*)	-
Wheezing age 2 years (%)		
No	82.1 (3,358)	82.6 (3,827)
Yes	17.9 (731)	17.4 (807)
*Missing*	*11*.*8* (*545*)	-
Wheezing age 3 years (%)		
No	89.0 (3,417)	89.4 (4,143)
Yes	11.0 (421)	10.6 (491)
*Missing*	*17*.*2* (*796*)	-

Values are percentages (absolute values), means (SD)* or medians (5-95^th^ percentile)^$^.

Missing percentages are given for the total population of analysis n = 4634. Other percentages are valid percentages.

### Air pollution and risk of wheezing

We observed no associations of average PM_10_ and NO_2_ concentrations during the previous year with the risks of wheezing at the ages of 1, 2 or 3 years separately or in the overall longitudinal model (Table 2). Additional analyses showed that children exposed to the highest 25% PM_10_ and NO_2_ levels did not have an increased risk of wheezing in the first 3 years compared to those exposed to the lowest 25% air pollutants levels (results not shown). At the age of 1 year only, information about the average exposure to air pollutants and wheezing during the last month was available. As compared to the average per year exposure we observed a larger variation in exposure levels of air pollutants measured in the previous month at the age 1 year (Additional file 1: Table S2). Furthermore, exposure to increased levels of PM_10_ during the previous month tended to be associated with an elevated risk of wheezing but the effect estimate did not reach statistical significance (OR 1.25 (0.98, 1.58) per 10 μg/m^3^). Increased levels of NO_2_ during the previous month were associated with wheezing (OR 1.32 (1.11, 1.55) per 10 μg/m^3^) (Table 3). We observed no time-dependent effect of air pollutants on wheezing in the first 3 years (p-values for interaction time*air pollutant: >0.05). We explored the confounding and modifying effect of lower respiratory tract infections and did not observe changes in our effect estimates after adjusting the analyses for lower respiratory tract infections. Also, the interaction between air pollution and lower respiratory tract infections was not significant, and we observed no associations between air pollutants and lower respiratory tract infections (data not shown).

**Table 2:** Exposure to air pollutants (previous year, overall) and risks of wheezing

	**Odds ratio of wheezing (95% Confidence Interval)**			
	**Age 1 year**	**Age 2 years**	**Age 3 years**	**Overall**
**PM**_**10**_				
Crude	1.07 (0.77, 1.50)	1.54 (0.90, 2.61)	1.00 (0.51, 1.95)	1.28 (0.85, 1.91)
Adjusted	1.21 (0.84, 1.74)	1.49 (0.83, 2.66)	0.90 (0.43, 1.91)	1.28 (0.83, 1.98)
**NO**_**2**_				
Crude	1.01 (0.85, 1.20)	1.04 (0.85, 1.27)	1.03 (0.79, 1.33)	1.05 (0.92, 1.19)
Adjusted	1.07 (0.89, 1.29)	1.04 (0.83, 1.29)	0.97 (0.72, 1.30)	1.07 (0.93, 1.23)

Values are odds ratios (95% confidence interval) from logistic regression models representing the risks of wheezing per 10 μg/m^3^ increase in PM_10_ or NO_2_. The overall effect is from generalized estimating equation models, based on average air pollution levels from birth until the age of 3 years with wheezing at the ages of 1, 2 and 3 years combined.

Models are adjusted for maternal age, education, parity, smoking, smoking of the partner, history of asthma or atopy and children’s sex, gestational age, birth weight, ethnicity, breastfeeding, daycare attendance, pet keeping and lower respiratory tract infections at the corresponding ages.

**Table 3:** Exposure to air pollutants in the previous month and wheezing in the same month

	**Odds ratio of wheezing in previous month age 1 year (95% Confidence Interval)**	
	**PM**_**10**_	**NO**_**2**_
	**n = 373**	**n = 373**
Quartile 1	Reference	Reference
	n = 83	n = 72
Quartile 2	1.24 (0.90, 1.71)	1.28 (0.91, 1.79)
	n = 97	n = 87
Quartile 3	1.08 (0.77, 1.49)	1.54 (1.11, 2.13)*
	n = 82	n = 103
Quartile 4	1.38 (1.01, 1.88)*	1.62 (1.17, 2.24)**
	n = 111	n = 111
Trend	1.25 (0.98, 1.58)	1.32 (1.11, 1.55)
	p = 0.07	p < 0.01

Values are odds ratios (95% confidence interval) for wheezing from logistic regression models. *P < 0.05 and **p < 0.01. Models are adjusted for maternal age, education, parity, smoking, smoking of the partner, history of asthma or atopy and children’s sex, gestational age, birth weight, ethnicity, breastfeeding, daycare attendance, pet keeping and lower respiratory tract infections at age 1 year. Trend represents the risk of wheezing per 10 μg/m^3^ increase in PM_10_ or NO_2_.

### Air pollution, tobacco smoke exposure and risk of wheezing

**Figure 2:**
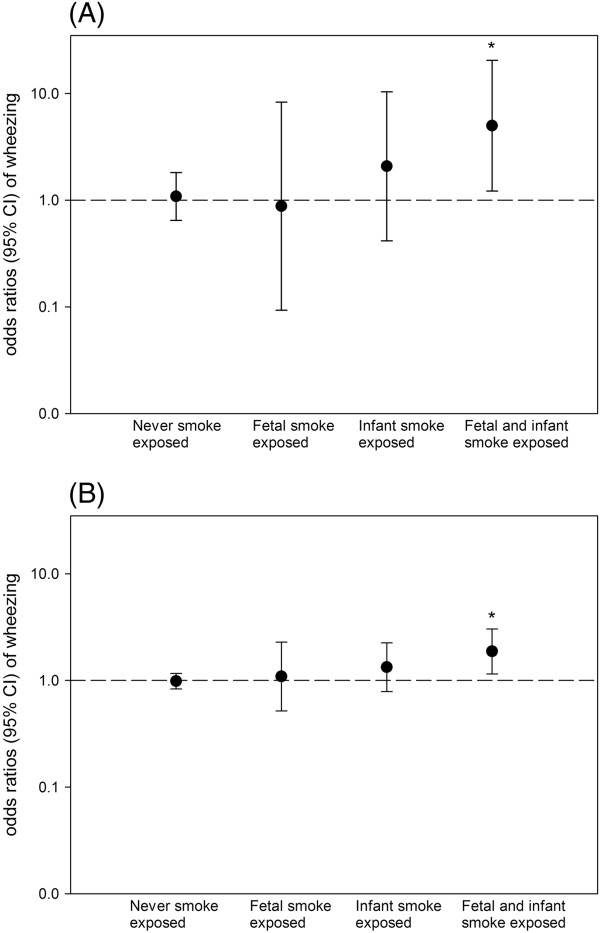
**Exposure to air pollutants PM**_**10**_**(A), NO**_**2**_**(B), tobacco smoke and wheezing.** Values are overall odds ratios (95% confidence interval) from generalized estimating equation models based on average air pollution levels from birth until the age of 3 years with wheezing at the ages of 1, 2 and 3 years combined, representing the risks of wheezing per 10 μg/m^3^ increase in PM_10_ or NO_2_ stratified for tobacco smoke exposure * p <0.05. Models are adjusted for maternal age, education, parity, history of atopy or asthma and children’s ethnicity, sex, gestational age, birth weight, breastfeeding, daycare attendance, pet keeping and lower respiratory tract infections at 1, 2 and 3 years of age. P-values for interaction: tobacco smoke exposure * average level PM_10,_ p-value <0.05; tobacco smoke exposure * average level NO_2,_ p-value <0.01.

We found no associations of air pollutants levels with the annual risks of wheezing stratified for fetal and infant smoke exposure (Additional file 1: Table S3). Stratified longitudinal analyses showed that the associations of average PM_10_ and NO_2_ exposure levels with the overall longitudinal risks of wheezing during the first 3 years of life were stronger and significant among children who were exposed to tobacco smoke both during fetal and infant life (overall odds ratios 4.54 (1.17, 17.65) and 1.85 (1.15, 2.96) per 10 μg/m^3^ increase in PM_10_ and NO_2_, respectively) (Figure 2). We did not observe associations of traffic-related air pollutants with wheezing among children who were exposed to smoke during fetal life only or during infancy only. However, we observed elevated odds ratios for infant smoke exposure, but these effect estimates were not significant. We additionally assessed whether tobacco smoke exposure modified the association of air pollution with risks of wheezing by using interaction terms. These interaction terms were statistically significant for the associations of air pollutants with longitudinally measured wheezing (P-values for interaction: PM10*smoking: p-value <0.05; NO2*smoking: p-value <0.01). However, per year analyses showed that the association of air pollutants with wheezing was modified by tobacco smoke exposure only at the age of 3 years (P-values for interaction per year: PM_10_*smoking: p-value = 0.35 (age 1), p-value = 0.20 (age 2), and p-value <0.05 (age 3). P-values for interaction NO_2_*smoking are: p-value = 0.23 (age 1), p-value = 0.14 (age 2), and p-value <0.05 (age 3)).

## Discussion

Our study suggests that long term exposure to higher levels of traffic-related air pollutants PM_10_ and NO_2_ are associated with increased risks of wheezing in the first 3 years of life among children who are exposed to tobacco smoke during fetal and infant life. We did not observe associations of traffic-related air pollutants with wheezing among children who were not exposed to tobacco smoke.

Previous studies reported inconsistent findings for the associations of traffic-related air pollution with asthma symptoms and doctor diagnosed asthma [6,7]. Associations of NO_2_ and PM_2.5_ with overall wheezing until the age of 8 years were observed in another study in the Netherlands [14]. A Swedish cohort study observed associations of air pollution in the first year of life with persistent wheezing until 4 years of age [25]. A study in Germany observed no associations of long term exposure to PM_2.5_ or NO_2_ with the risks of parental reports of asthma symptoms, but observed an association of PM_2.5_ exposure levels with doctor diagnosed asthma at the age of 6 years [26]. Finally, a large Canadian study reported inconsistent results for the associations of air pollutant levels with the risk of asthma until the age of 4 years, depending on the exposure assessment. The authors reported no association of traffic-related air pollution based on land use regression modeling with the risks of asthma, but reported associations of distance to industrial point sources with an increased risk of asthma [27]. Differences between our study and previous published studies include our detailed method to assess air pollution exposure levels in a large city, the availability of many potential confounders and the interaction with smoke exposure. Also, earlier studies did not use individual exposure levels [27], took only the birth addresses into account or were not able to adjust for home movement [9,14,25]. Children in our study were exposed to a smaller range of NO_2_ exposure (range 28.8-56.1 μg/m^3^) as compared with another Dutch study (NO_2_ range 12.6-58.4 μg/m^3^) which might have led to smaller effect estimates [14]. By using long term exposure averages, the potential short term high risk exposure levels may be missed. At the age of 1 year only, we obtained information about wheezing in the last month and the average exposure to air pollutants during that month. Increased levels of air pollutants exposure during the previous 1 month were associated with increased risks of wheezing. We were not able to assess this short time interval at older ages.

We observed an interaction between air pollution and tobacco smoke exposure for the association with longitudinally measured wheezing. However, in our per year analyses we observed that this interaction was only significant at the age of 3 years. This might be explained by the idea that from the age of 3 years onwards wheezing represents another phenotype than earlier wheezing in which other factors such as atopic susceptibility in the origins of wheezing become more important. Also, infant smoke exposure was assessed after respiratory outcomes at age 1 year. This might be a reason for observing no significant interaction between exposure to air pollutants, tobacco smoke and wheezing before the age of 3 years. Our results suggest that tobacco smoke exposure increases the vulnerability of the lungs to air pollutants. The interaction between particulate matter and tobacco smoke exposure was previously explored by Rabinovitch et al. [3]. They observed that environmental tobacco smoke exposure modifies the acute effects of low-level ambient PM_2.5_ exposure on childhood asthma. Albuterol usage and leukotriene E_4_ were only related to PM_2.5_ concentrations on days when urine cotinine levels were low, which suggest that only when children were not or to a small amount exposed of tobacco smoke, exposure to air pollution was positively associated with asthma. Their results were in the opposite direction as compared to our results. This difference might be explained by differences in study design and methods. We assessed reported tobacco smoke exposure both in fetal and infant life, wheezing at younger ages, and long term exposure to tobacco smoke and air pollution. Rabinovitch et al. assessed biological markers of smoke exposure in childhood, used albuterol usage as a proxy for asthma, at an older age, and assessed the short term effects of air pollutants. Previous studies suggested that both short term and long term exposure to air pollutants are important for the development of asthma exacerbations or respiratory symptoms [25,28-34]. Our results suggest that short term exposure to air pollutants might be important for developing respiratory symptoms, whereas long term exposure to air pollutants might be important in the presence of tobacco smoke exposure. However our results should be considered as hypothesis generating. More studies are needed to explore the combined effects of air pollution and tobacco smoke exposure on the development of respiratory symptoms. Previously, we have reported that children from mothers who smoked continuously during pregnancy and during the first years after pregnancy had increased risks of wheezing in the first years of life [15]. Fetal smoke exposure has been suggested to have a different underlying mechanism in the pathway to wheezing than infant smoke exposure. Fetal smoke exposure may lead to impaired lung development and immunological changes while for infant smoke exposure it includes bronchial hyperreactivity, immunological changes, and direct toxic and irritant effects [35-37]. Increased vulnerability of the airways and lungs to air pollutants might be caused by both fetal and infant smoke exposure via their pathophysiological mechanisms. Among children with infant smoke exposure, we observed a non-significant elevated odds ratio for the associations of air pollution with wheezing. This tendency was not observed in children with only fetal smoke exposure. This might be due to the direct toxic effects of both infant smoke exposure and exposure to air pollutants, which are absent in fetal smoke exposure only [38]. The mechanisms underlying the association of air pollution exposure with wheezing or asthma might also include the induction of airway inflammation and oxidative stress, modification of enzyme functions, disruption of immune responses and increased reactivity to allergens [26,38-40]. Also, respiratory infectious diseases might play a role. However, we did not observe a confounding or modifying effect of respiratory tract infections or associations between air pollutants and respiratory tract infections. Therefore, the associations of air pollution with wheezing in our study are probably not explained by infectious mechanisms. Further studies exploring potential underlying causal mechanisms are needed.

This study was embedded in a population-based prospective design with a large number of subjects being studied from early life onwards with detailed and frequently prospectively measured information about air pollution levels at the corresponding home-addresses. We adjusted for a large number of confounders and the results did not differ between non-imputed and imputed analysis. Non-response at enrolment and lost to follow-up would lead to biased effect estimates if the associations of air pollutants with wheezing would be different between those included and not included in the analyses. Selection bias due to non-participation at enrolment in the prenatal phase might have occurred because our study population tends to have a selection towards more affluent and healthy mothers [16] who might have reported less wheezing symptoms and tobacco smoke exposure in their children and have been exposed to lower air pollutant levels [41]. If so, our observed effect estimates would be underestimated. Mothers and children lost to follow-up during the postnatal phase were lower educated (67% vs. 47%) and smoked more frequently during pregnancy (21% vs. 13%). If children who were lost to follow up would have had more wheezing episodes, this could have led to an underestimation of the observed effect of air pollution and tobacco smoke exposure on wheezing as well. One of the limitations of our study is that we might reflect a selection towards a more healthy population, as the prevalence of preterm birth is lower than average in The Netherlands, 4.7% versus 7.7%. A homogeneous population would not affect the observed association of air pollution with wheezing among children exposed and not exposed to tobacco smoke. However such a population might affect the generalizability. The observed effects might be different in a population with more preterm born children. Also, preterm birth could modify the effect between air pollution and wheezing, because airways and lungs of preterm born children might be less developed and therefore might be even more vulnerable to air pollution. Previous studies were limited in their ability to consider the intraurban gradients and temporal variations in air pollutants. However, some had obtained more subject-specific exposure levels [6,7]. A strength of our study is that we were able to consider detailed spatial and temporal contrasts in exposure, in which we were able to take home movements into account. In the first 3 years of life 39.9% of the children moved at least once. Still there might be misclassification of air pollution assessment. We only calculated exposure levels at home addresses and not at the day care centers or other places where the child may spend days and nights. We assumed that most of the time children until the age of 3 years are near or at their home addresses. Furthermore, other types of indoor or commuting exposure were not taken into account. If any, we expect that this misclassification is non-differential and may have led to an underestimation of the associations [42]. We had no information on smaller particle sizes than 10 μm. Smaller particles sizes such as PM_2.5_ might more adversely affect respiratory morbidity than PM_10_ due to deeper peripheral lung deposition. However, previous studies which measured both PM_10_ and PM_2.5_ observed strong correlations between exposure to PM_10_ and PM_2.5_ and similar effect sizes of these exposures on childhood asthma or wheezing [32,43]. Although assessing smoking habits by questionnaires is valid in epidemiological studies, misclassification may occur due to underreporting [44]. However, the use of biomarkers of tobacco smoke exposure in urine, saliva or blood, or nicotine in indoor air seems not superior to self-report [44-47]. First trimester adverse exposures might be important for fetal lung development [48]. Using data from the same study population, we have previously shown that children do not have an increased risk of preschool wheezing when mothers quitted smoking as soon as they knew they were pregnant [15]. Based on results of our previous study, we categorized no fetal smoke exposure as children who were never exposed to tobacco smoke or were exposed to tobacco smoke until first trimester of pregnancy only [15]. We performed a sensitivity analysis without including fetal smoke exposure during first trimester only, and observed that the effect sizes did not materially change. Still, it might be that our categorization led to some misclassification, with an underestimation of the effect estimates when first trimester only smoking would have comparable effects as continued smoking during pregnancy. The main outcome in our study was self-reported wheezing. This method is widely accepted in epidemiological studies and reliably reflects the prevalence of wheezing in young children [49]. In preschool children a diagnosis of asthma is based on symptoms [50], and objective tests, including lung function or bronchial responsiveness, are difficult to perform in young children and have a very limited if any diagnostic value. Follow up studies at older ages will include more detailed asthma and atopy measurements.

## Conclusions

In conclusion, our results suggest that higher long term exposure levels to traffic-related air pollution lead to higher risks of wheezing in preschool children who were exposed to fetal and infant tobacco smoke. Further studies are needed to explore underlying mechanisms of exposure to air pollutants with and without interaction with tobacco smoke exposure and various types of wheezing and asthma in later life.

## Abbreviations

GEE model: Generalized Estimating Equation model; OR: Odds ratio; 95% CI: 95% confidence interval; PM_10_: Particulate matter ≤10 μm; NO_2_: Nitrogen dioxide; SD: Standard deviation.

## Competing interests

The authors declare that they have no competing interests.

## Authors' contributions

AS, YK, VJ, JJ and LD contributed to the conception and design, acquisition of data, analyses and interpretation of the data, drafted the article, revised it critically for important intellectual content and gave final approval of the version to be published. CG, HR, HM, FP, AH and HM contributed to the conception and design and acquisition of data, revised it critically for important intellectual content and gave final approval of the version to be published.

## Supplementary Material

Additional file 1 **The following supplementary tables are available.Table S1.** Cross table of fetal smoke exposure with infant smoke exposure. **Table S2.** Levels of air pollutant. **Table S3.** Exposure to air pollutants in previous year, tobacco smoke and wheezing. Click here for file
