# Supplementary material for: Air pollution, fetal and infant tobacco smoke exposure, and wheezing in preschool children: a population-based prospective birth cohort
Source: Environ Health. 2012 Dec 11;11:91. doi: 10.1186/1476-069X-11-91 (PMC3533997; doi:10.1186/1476-069X-11-91)
Supplement: Additional file 1 — The following supplementary tables are available.Table S1. Cross table of fetal smoke exposure with infant smoke exposure. Table S2. Levels of air pollutant. Table S3. Exposure to air pollutants in previous year, tobacco smoke and wheezing. [file 1476-069X-11-91-S1.pdf]

Air pollution, fetal and infant tobacco smoke exposure, and wheezing in preschool children: a population-based prospective birth cohort.

Agnes M.M. Sonnenschein-van der Voort, MSc, Yvonne de Kluizenaar, MSc,

Vincent W.V. Jaddoe, MD, PhD, Carmelo Gabriele MD, PhD, Hein Raat, MD, PhD,

Henriëtte A. Moll, MD, PhD, Albert Hofman, Frank H. Pierik, PhD, MD, PhD,

Henk M.E. Miedema, PhD, Johan C. de Jongste, MD, PhD, Liesbeth Duijts, MD, PhD

Table S1. Cross table of fetal smoke exposure with infant smoke exposure

	No infant smoke exposure (%)	Infant smoke exposure (%)	*Total*
No fetal smoke exposure (%)	3,513 (87.8)	490 (12.2)	4,003 (100)
Fetal smoke exposure (%)	257 (40.7)	374 (59.3)	631 (100)
Total	3,770	864	4,634

Values are numbers (percentages)

Table S2. Levels of air pollutant

	Overall	Previous month	Previous year		
	**Age 1 - 3 years**	**Age 1 year**	**Age 1 year**	**Age 2 years**	**Age 3 years**
**PM_{10} ($\mu g/m^3$)**	n=3,295	n=3,898	n=3,963	n=3,771	n=3,166
Mean (SD)	28.36 (1.29)	28.29 (4.61)	28.86 (2.11)	28.27 (1.57)	27.92 (1.67)
Min	25.84	20.04	24.47	24.19	23.96
25%	27.49	24.77	27.49	27.29	26.73
50%	28.18	27.51	28.60	28.25	27.91
75%	28.89	31.59	29.78	29.13	28.91
Max	36.01	44.28	39.81	35.82	35.76
**NO_2 ($\mu g/m^3$)**	n=3,295	n=3,897	n=3,963	n=3,772	n=3,166
Mean (SD)	37.39 (4.01)	38.14 (6.81)	38.66 (4.20)	37.46 (4.17)	36.22 (4.28)
Min	28.81	18.20	29.66	27.10	27.02
25%	34.61	33.73	35.72	34.54	33.35
50%	37.10	39.07	38.34	37.33	35.69
75%	39.32	42.95	40.68	39.49	38.58
Max	56.05	58.27	59.60	55.87	55.68

Table S3. Exposure to air pollutants in previous year, tobacco smoke and wheezing

	Odds ratio of wheezing (95% CI)									
	PM_{10}					NO_2				
		Tobacco smoke exposure					Tobacco smoke exposure			
	Total	Never	Fetal	Infant	Fetal- and infant	Total	Never	Fetal	Infant	Fetal- and infant
Age 1 year	1.21 (0.84, 1.74)	1.09 (0.71, 1.68)	1.38 (0.24, 7.97)	2.22 (0.65, 7.59)	1.96 (0.50, 7.64)	1.07 (0.89, 1.29)	1.00 (0.81, 1.24)	1.35 (0.53, 3.45)	1.32 (0.67, 2.60)	1.49 (0.75, 2.97)
Age 2 years	1.49 (0.83, 2.66)	1.29 (0.65, 2.54)	0.57 (0.04, 9.39)	3.98 (0.54, 29.59)	4.40 (0.56, 34.40)	1.04 (0.83, 1.29)	0.97 (0.75, 1.26)	0.73 (0.25, 2.13)	1.32 (0.60, 2.88)	1.76 (0.84, 3.71)
Age 3 years	0.90 (0.43, 1.91)	0.59 (0.24, 1.43)	0.39 (0.01, 19.83)	4.07 (0.27, 60.76)	3.80 (0.36, 40.54)	0.97 (0.72, 1.30)	0.86 (0.60, 1.21)	0.40 (0.07, 2.20)	0.88 (0.30, 2.60)	2.34 (0.96, 5.67)

Values are odds ratios (95% confidence interval) for wheezing at the ages of 1, 2 and 3 years per 10 $\mu g/m^3$ increase of PM_{10} or NO_2 in the total group and stratified for fetal and infant tobacco smoke exposure. *$P < 0.05$. Models are adjusted for maternal age, education, parity, history of asthma or atopy and children's sex, gestational age, birth weight, ethnicity, breastfeeding, daycare attendance, pet keeping and lower respiratory tract infections at the corresponding ages. Total analyses were additionally adjusted for maternal smoking and smoking of the partner. P-values for interaction PM_{10} * smoking: p-value = 0.35 (age 1), p-value = 0.20 (age 2), and p-value <0.05 (age 3). P-values for interaction NO_2 * smoking: p-value = 0.23 (age 1), p-value = 0.14 (age 2), and p-value <0.05 (age 3).
